# Supplementary material for: Integrative epigenetic taxonomy of primary prostate cancer
Source: Nat Commun. 2018 Nov 21;9:4900. doi: 10.1038/s41467-018-07270-2 (PMC6249266; doi:10.1038/s41467-018-07270-2)
Supplement: Supplementary file 1 — Supplementary Information [file 41467_2018_7270_MOESM1_ESM.pdf]

Supplementary Information

Integrative epigenetic taxonomy of primary prostate cancer

Stelloo, Nevedomskaya, Kim et al.,

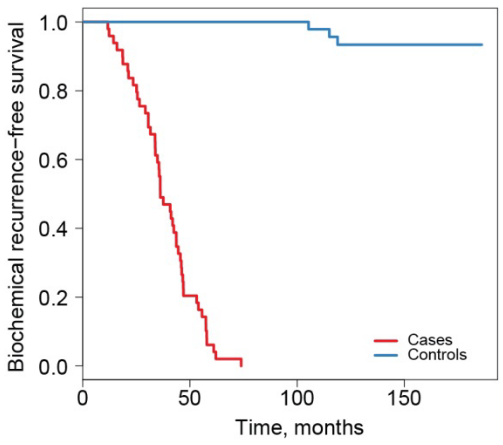

Supplementary Figure 1: Biochemical recurrence-free survival

Kaplan-Meier curve of biochemical recurrence-free survival between the two groups; cases and controls.

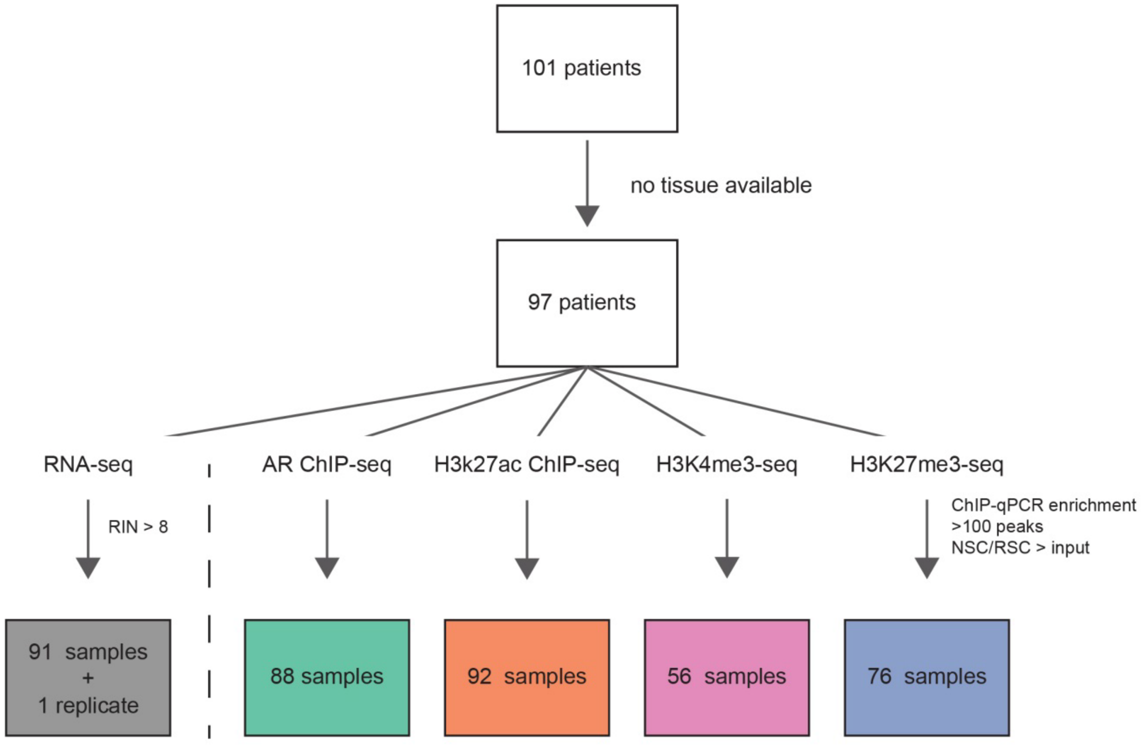

Supplementary Figure 2: Sample flow diagram

Flow diagram for sample quality control analyses, indicating numbers of samples passing the required cut-off values as defined for each datastream.

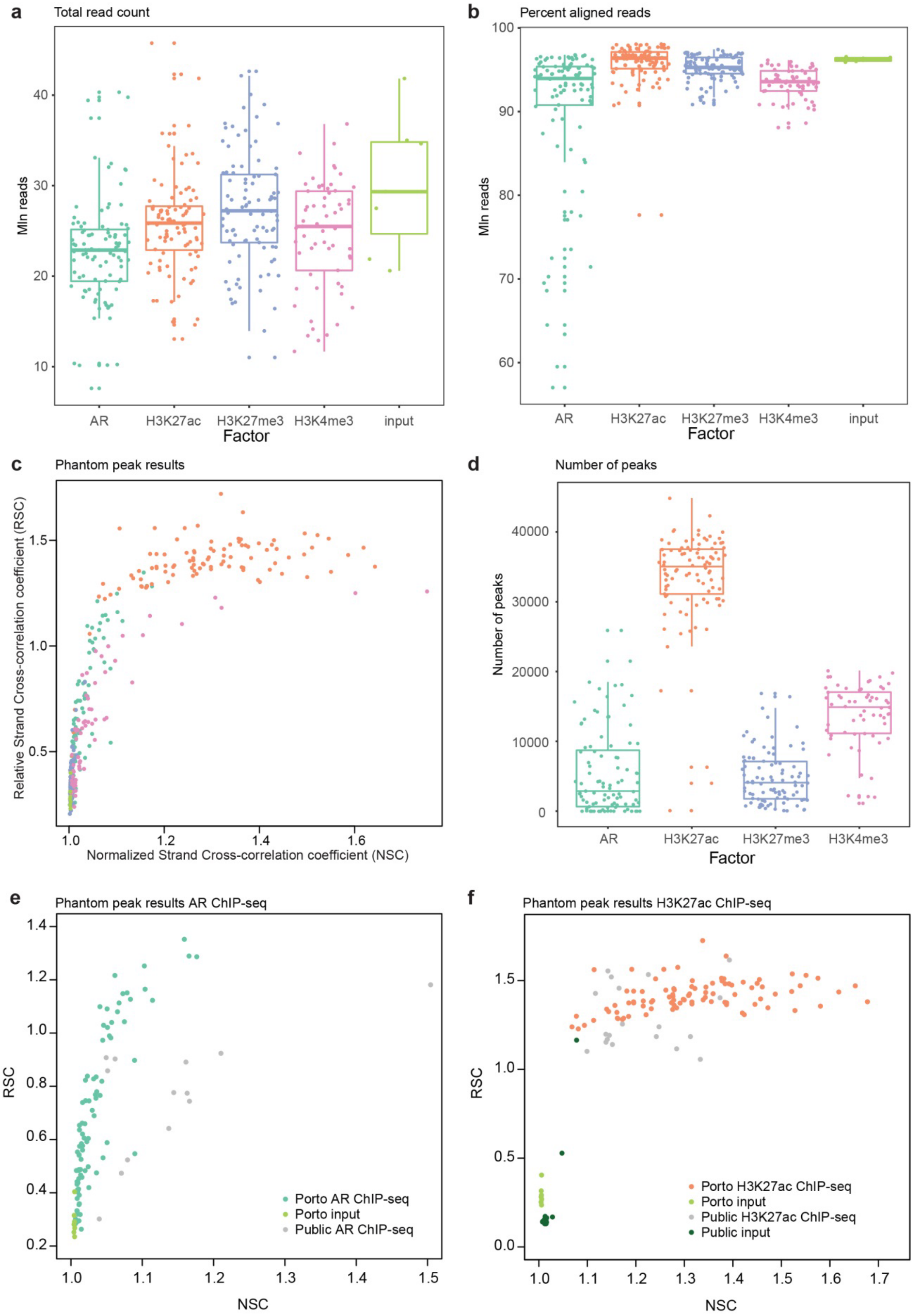

a
Total read count
Mln reads
Factor
AR
H3K27ac
H3K27me3
H3K4me3
input
b
Percent aligned reads
Mln reads
Factor
c
Phantom peak results
Relative Strand Cross-correlation coefficient (RSC)
Normalized Strand Cross-correlation coefficient (NSC)
d
Number of peaks
Number of peaks
Factor
e
Phantom peak results AR ChIP-seq
RSC
NSC
Porto AR ChIP-seq
Porto input
Public AR ChIP-seq
f
Phantom peak results H3K27ac ChIP-seq
RSC
NSC
Porto H3K27ac ChIP-seq
Porto input
Public H3K27ac ChIP-seq
Public input

Supplementary Figure 3: Number of reads, peaks and phantom peak results in ChIP-seq datasets of AR, H3K27ac, H3K27me3 and H3K4me3.

- **a.** Boxplots of the total number of reads in millions (mln). Boxplot: median values with interquartile range.
- **b.** Boxplots of the percentage of aligned reads per sample.
- **c.** Scatter plot of normalized strand cross-correlation coefficient (NSC) scores and relative strand cross-correlation coefficient (RSC) scores for all ChIP-seq samples and inputs.
- **d.** Number of peaks called by both peak callers for AR, H3K27ac and H3K4me3 ChIP-seq samples.
- **e.** Scatter plot of NSC scores RSC scores for inputs and AR ChIP-seq samples from this study and Pomerantz et al (GSE56288).
- **f.** Scatter plot of NSC scores and RSC scores for H3K27ac ChIP-seq samples and inputs from this study and Kron et al (EGAS00001002496).

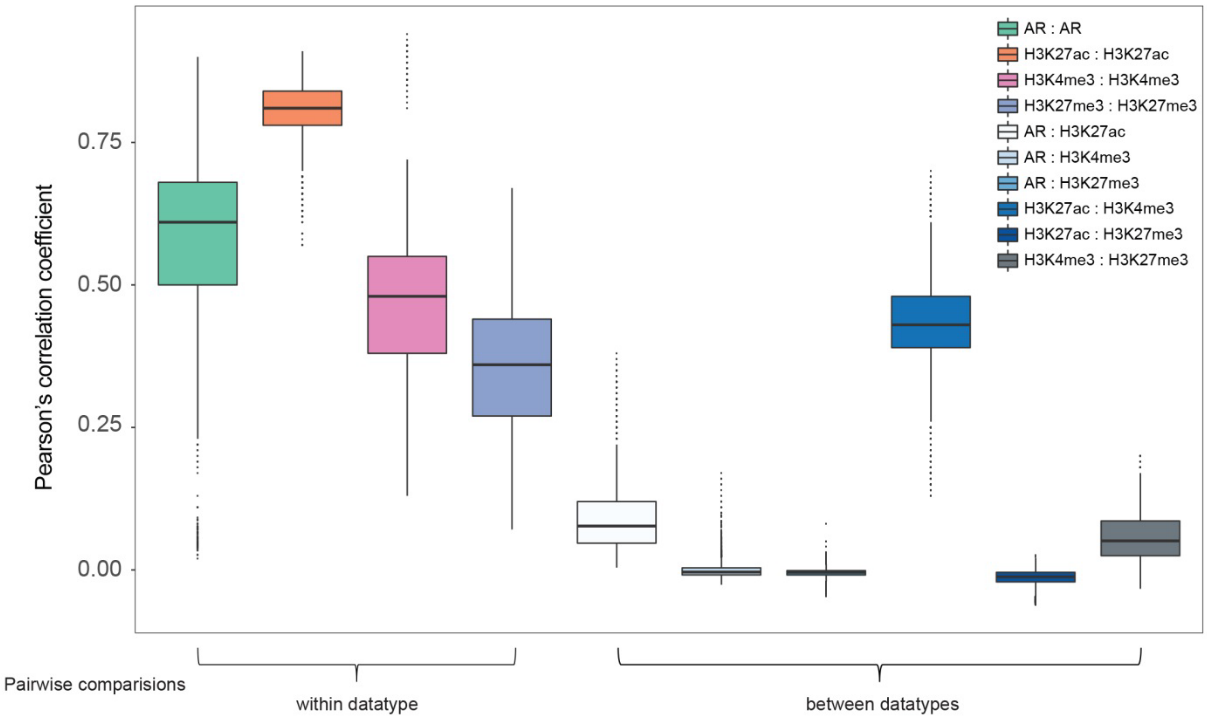

Supplementary Figure 4: Correlations between ChIP-seq samples

Boxplots showing the Pearson's correlation coefficients between ChIP-seq samples on the basis of called peaks. Center line, median; box limits, upper (75) and lower (25) quartiles; whiskers, 1.5x interquartile range. The correlation matrix is shown in Figure 2e.

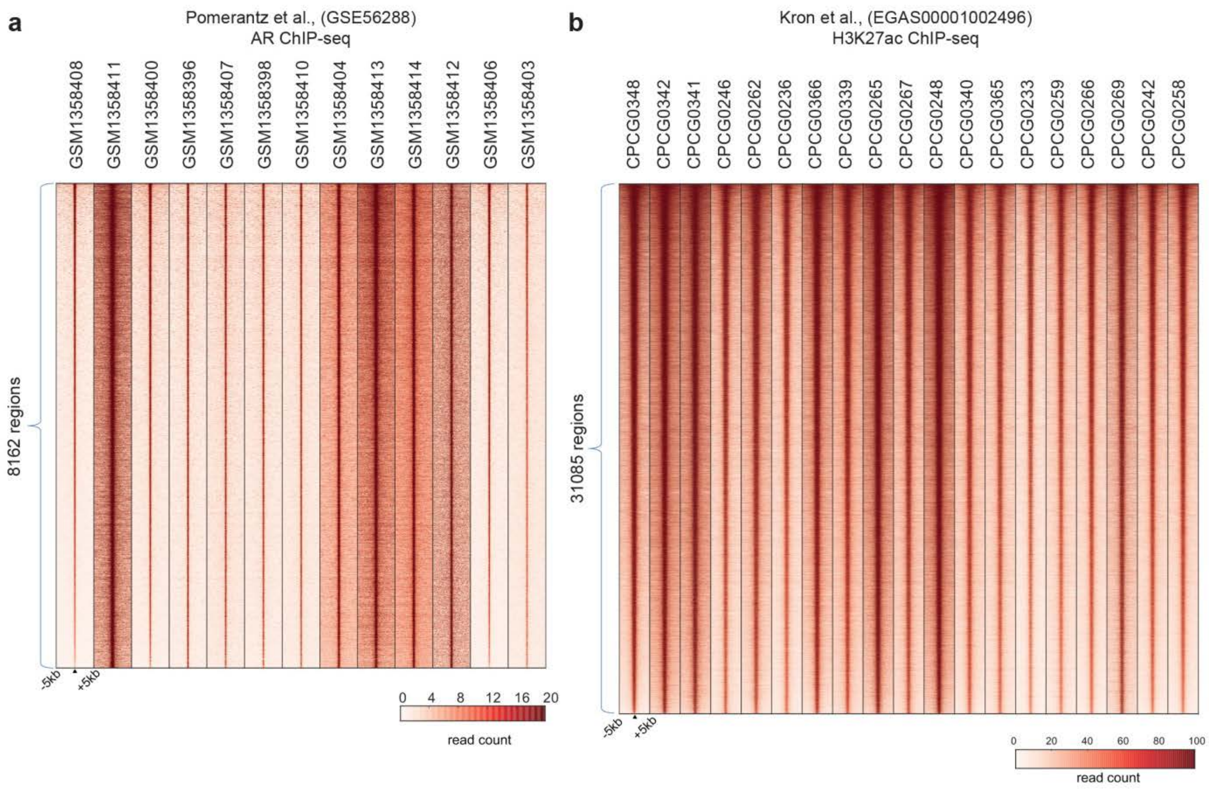

Supplementary Figure 5: Enrichment of AR and H3K27ac from publicly available data at the consensus sites

Heatmap of AR (a) and H3K27ac (b) ChIP-seq signal from publicly available datasets at the consensus peaksets defined on the Porto cohort. The following color scale is used: white, no enrichment; red, high enrichment.

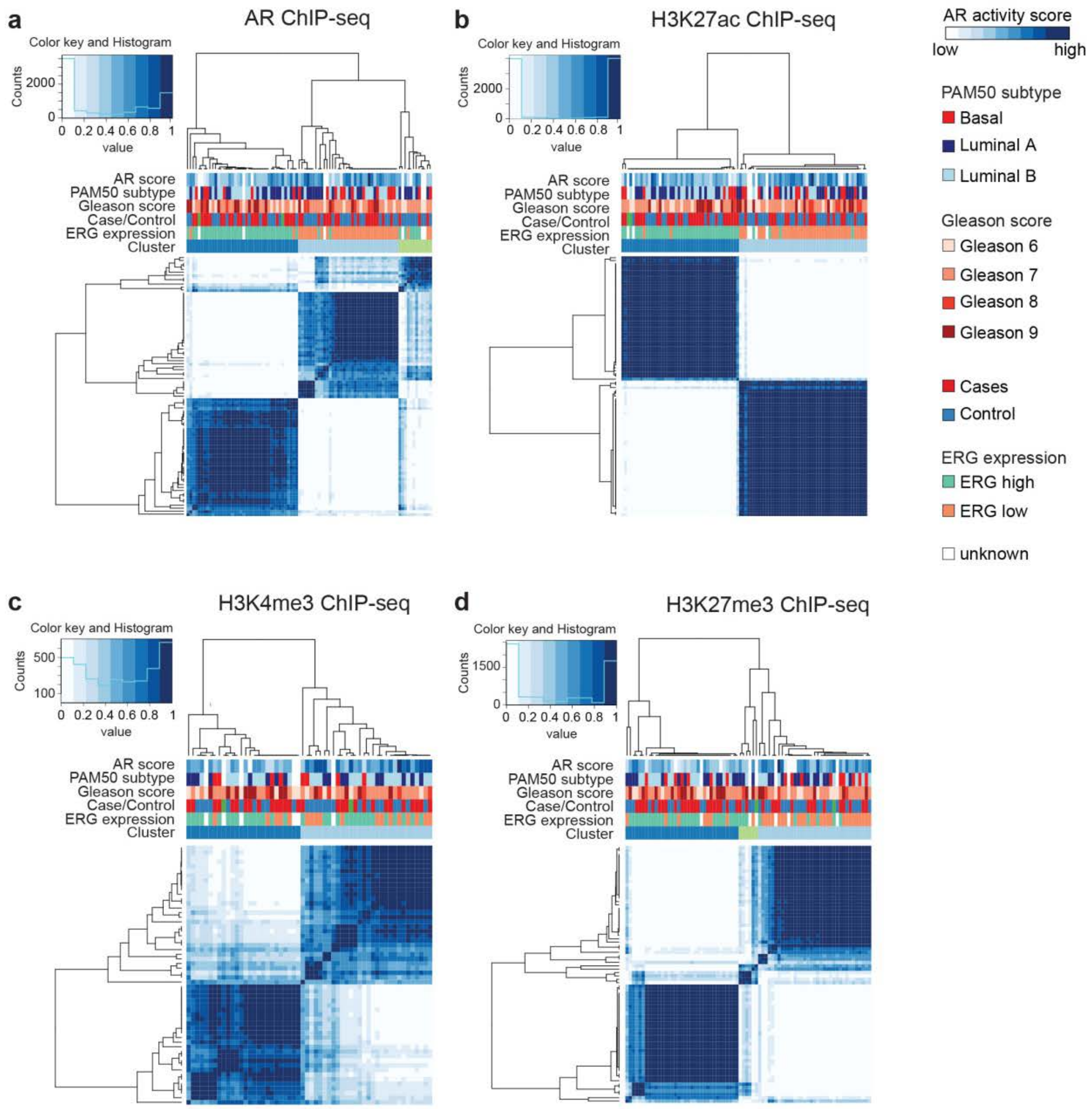

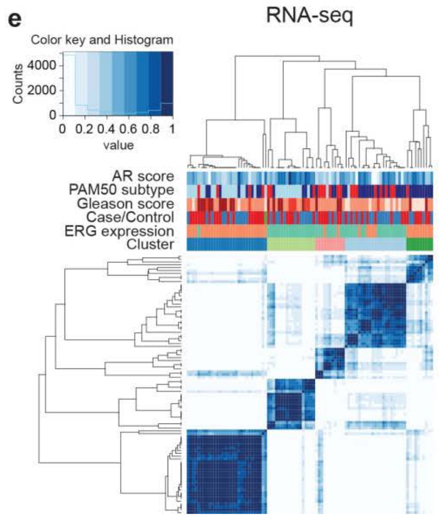

Supplementary Figure 6: Consensus clustering heatmaps

Consensus cluster analysis of AR ChIP-seq (a), H3K27ac ChIP-seq (b), H3K4me3 ChIP-seq (c), H3K27me3 ChIP-seq (d) and RNA-seq (e). The blue color depicts the frequency at which samples have been clustered together in permutations, the darker the color the higher the frequency of co-clustering. Each sample is annotated for AR activity score, PAM50 subtype, Gleason score, case/control status, ERG expression and cluster assignment.

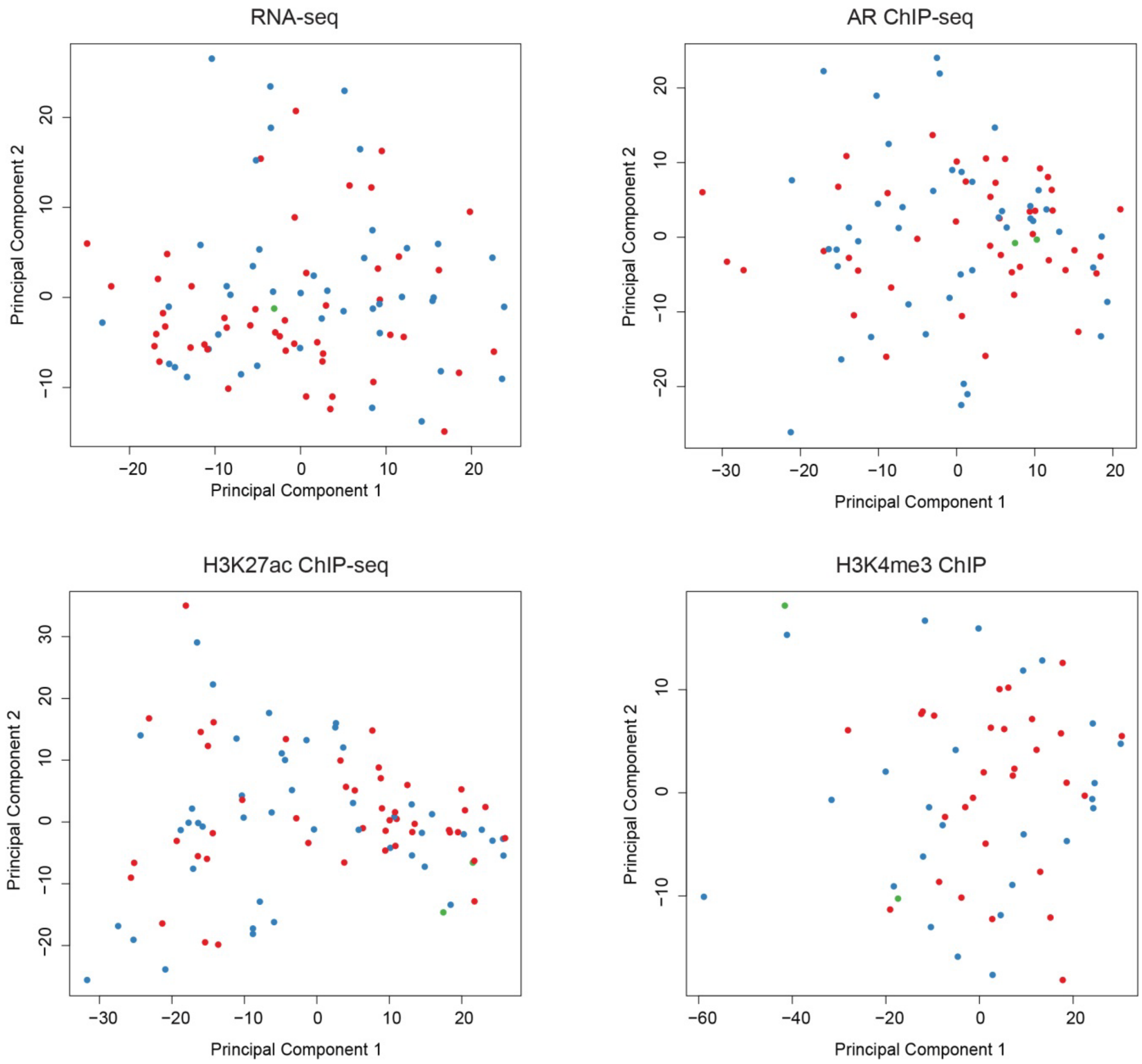

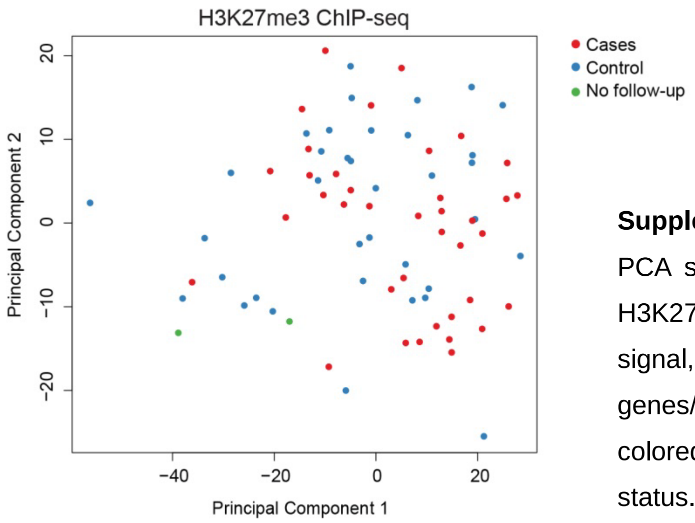

Supplementary Figure 7: PCA cases controls

PCA scores plot for RNA expression, AR binding, H3K27ac, H3K4me3 and H3K27me3 ChIP-seq signal, based on the top 1000 most-varying genes/regions across the samples. Samples are colored according to case (red)/ control (blue) status.

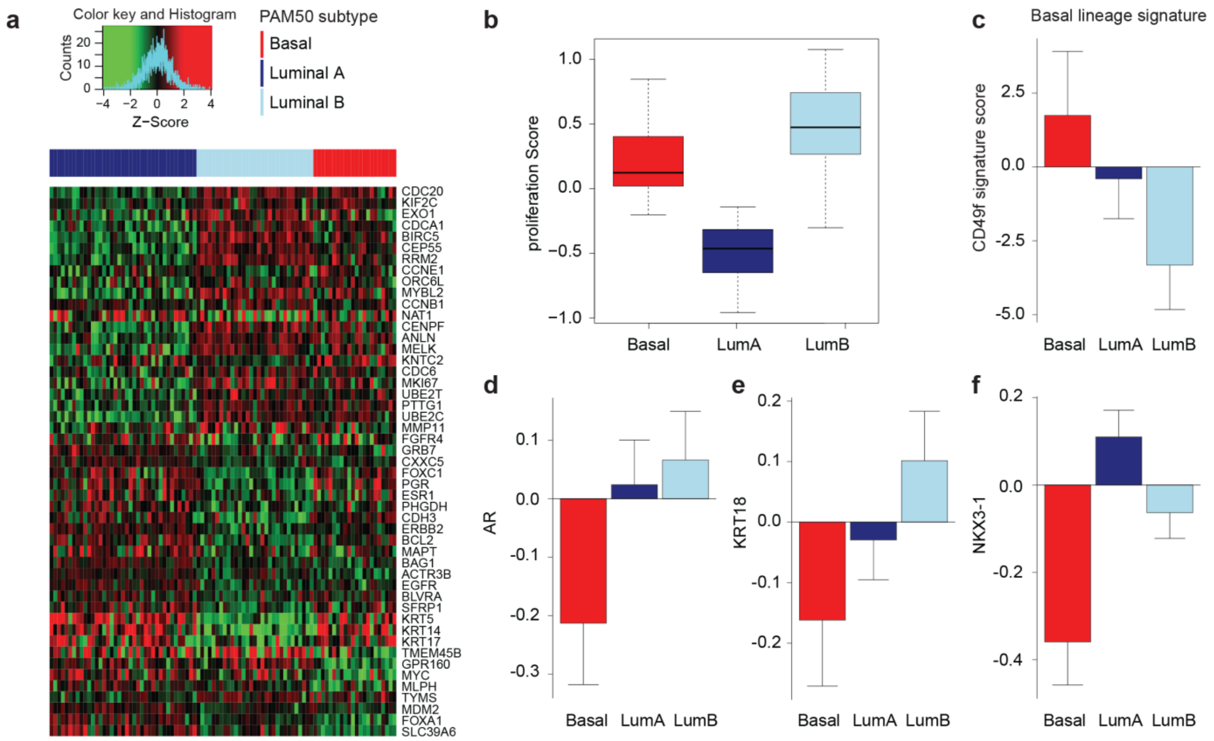

Supplementary Figure 8: PAM50 subtyping

- **a.** Heatmap depicting PAM50 classification for Luminal A (dark blue), Luminal B (light blue) or Basal (red) subtypes for each patient. The genes are ordered as shown in Zhao et al., 2017. Color scale: red indicates high expression an green low expression (z-score).
- **b.** Boxplot showing proliferation score across the three PAM50 subtypes. Center line, median; box limits, upper (75) and lower (25) quartiles; whiskers, 1.5x interquartile range.

c-f. Bar plots showing basal lineage CD49f signature score (**c**), AR expression (**d**), and expression of luminal lineage markers KRT18 (**e**) and NKX3-1 (**f**) across the three PAM50 subtypes. Mean (±SE) values of median centered log-transformed gene expression is shown.

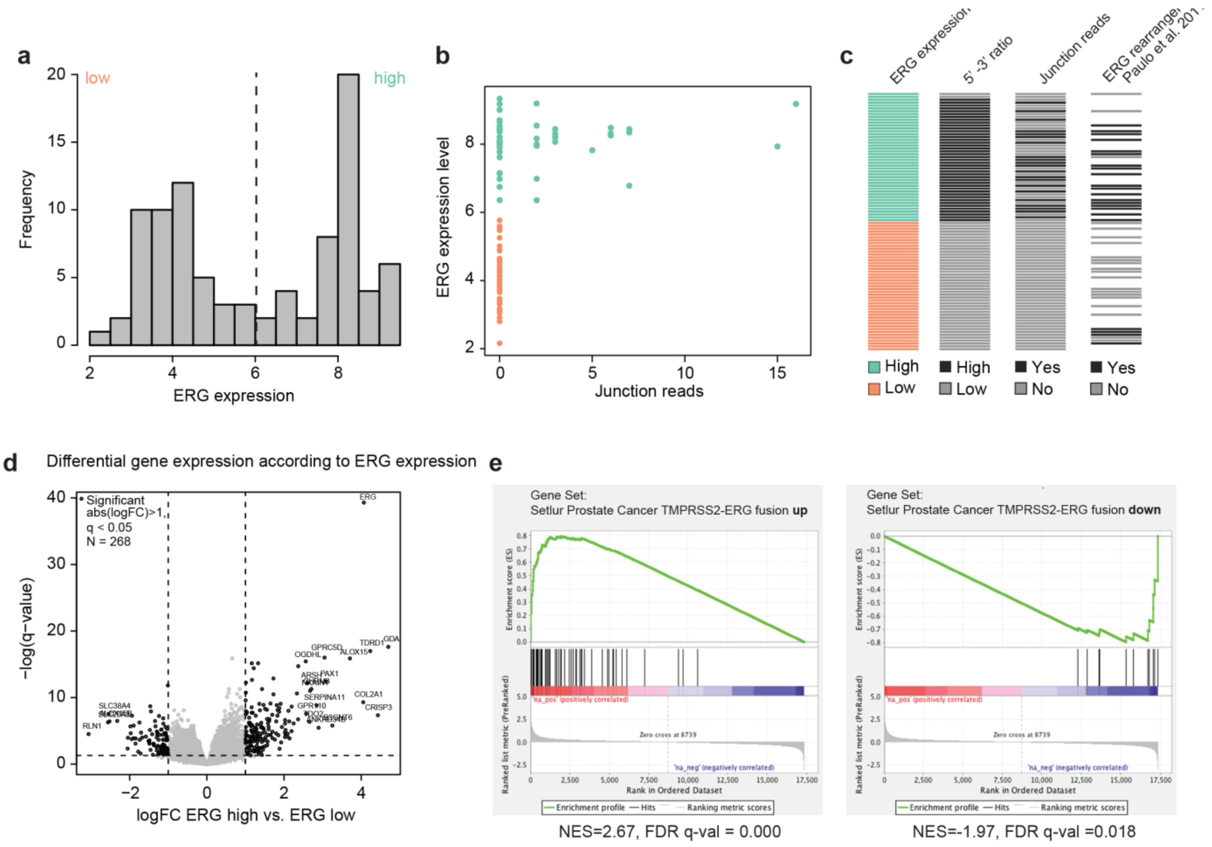

Supplementary Figure 9: ERG fusion status

- **a.** Distribution of ERG expression. The x-axis shows the log2 expression of ERG. The threshold between high and low ERG expression is shown by the dotted line.
- **b.** The number of fusion junction spanning reads are plotted on the x-axis and the log2 ERG expression on the y-axis.
- **c.** Summary of ERG fusion status per sample (rows) measured by either gene expression level, 5'–3' transcript ratio, the presence of fusion junction spanning reads and FISH/Taqman [12].
- **d.** Volcano plot showing differentially expressed genes between low ERG expressing tumors and high ERG expressing tumors. The negative log10 *p* values (y-axis) are plotted against the log2 fold change of expression (x-axis).
- **e.** Enrichment plots for MSigDB Setlur_prostate_cancer_tmprss2_erg_fusion_up and _down genesets.

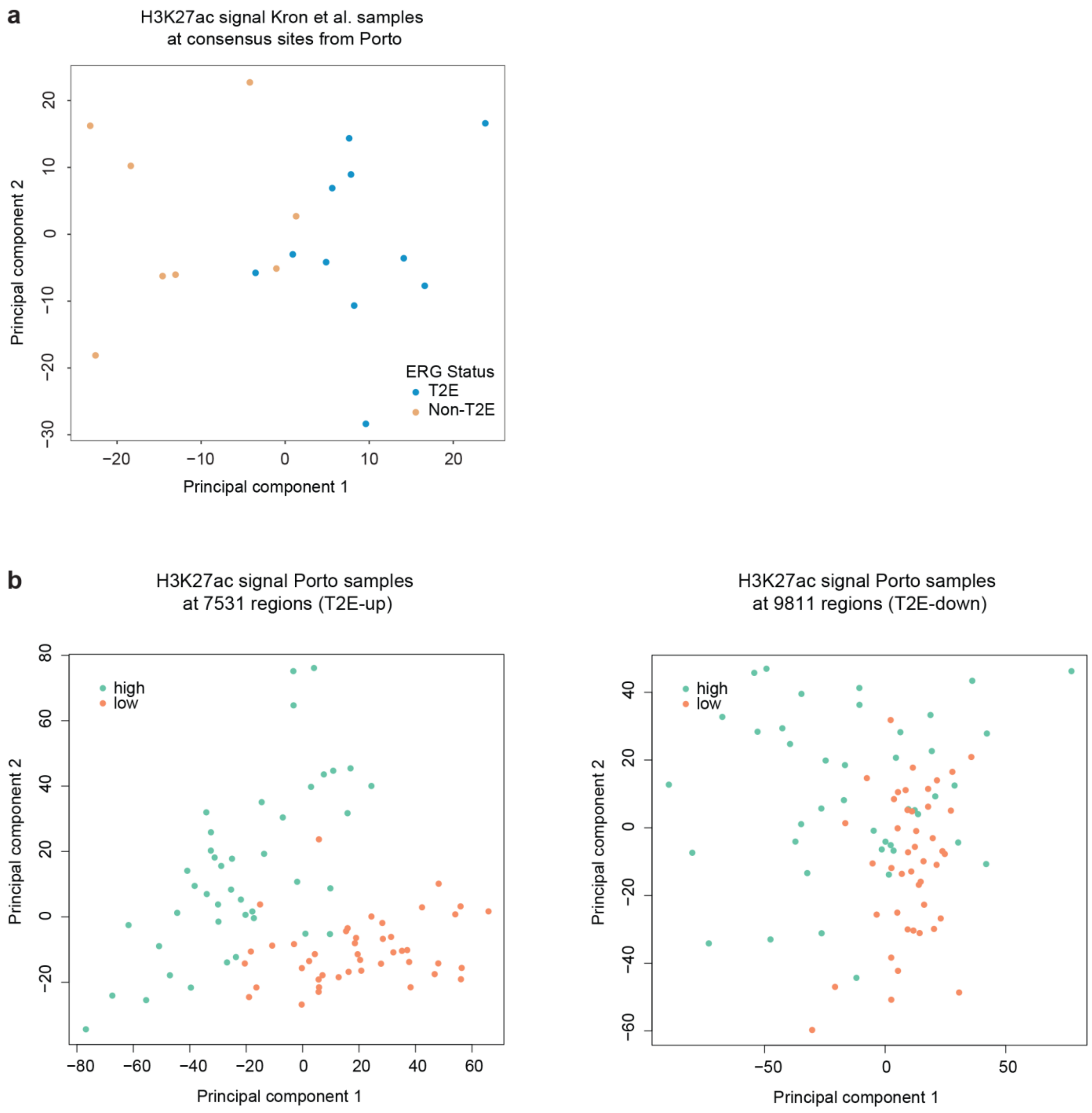

Supplementary Figure 10: Comparison of ChIP-seq data to discriminate samples on ERG status

a. PCA scores plot based on publicly available H3K27ac ChIP-seq signal at the 1000 regions defined in this study. Samples are colored according to ERG fusion status. (T2E: TMPRSS2-ERG fusion)

b. PCA scores plot for AR ChIP-seq signal (Porto samples) at the regions defined in the previous study, either 7531 regions (T2E-up) or 9811 region (T2E-down). Samples are colored according to ERG expression.

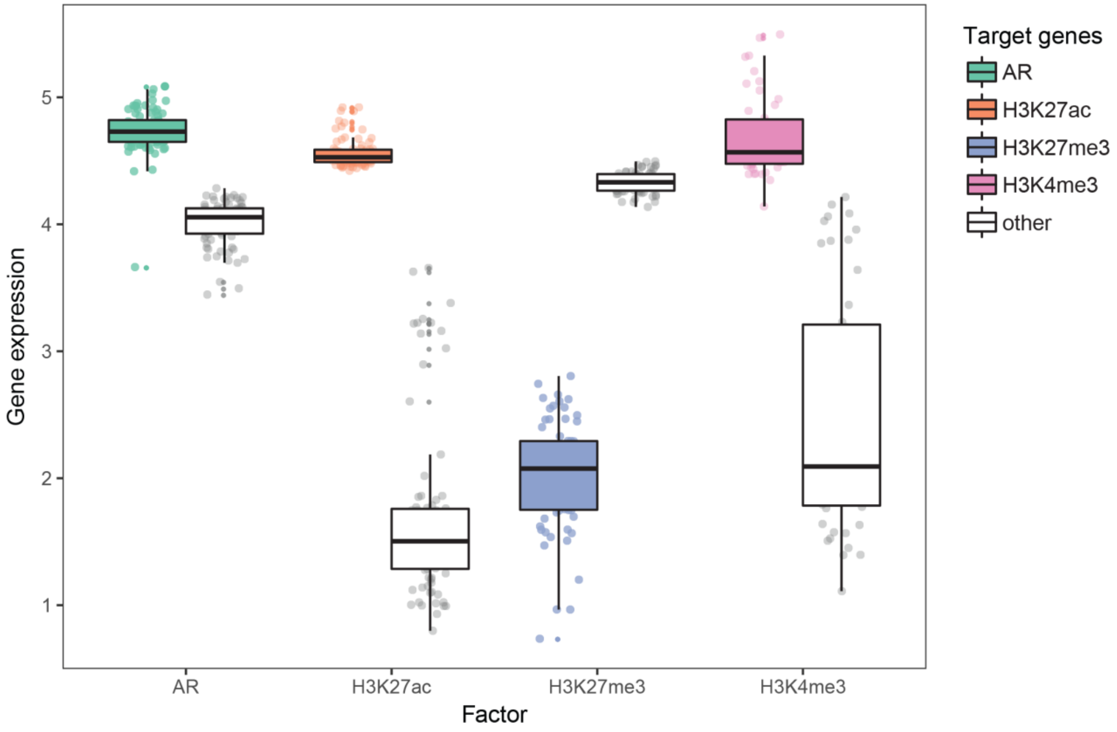

Supplementary Figure 11: Gene expression level of genes with proximal AR or H3K27ac, H3K27me3 and H3K4me3 chromatin marks.

Boxplots of per-patient average expression levels of potential target genes and the other genes (gray) for AR (green), H3K27ac (orange), H3K24me3 (purple), and H3K4me3 (pink). Target genes are defined as genes with called peak 20kb upstream of its transcription starting site and gene body. Center line, median; box limits, upper (75) and lower (25) quartiles; whiskers, 1.5x interquartile range.

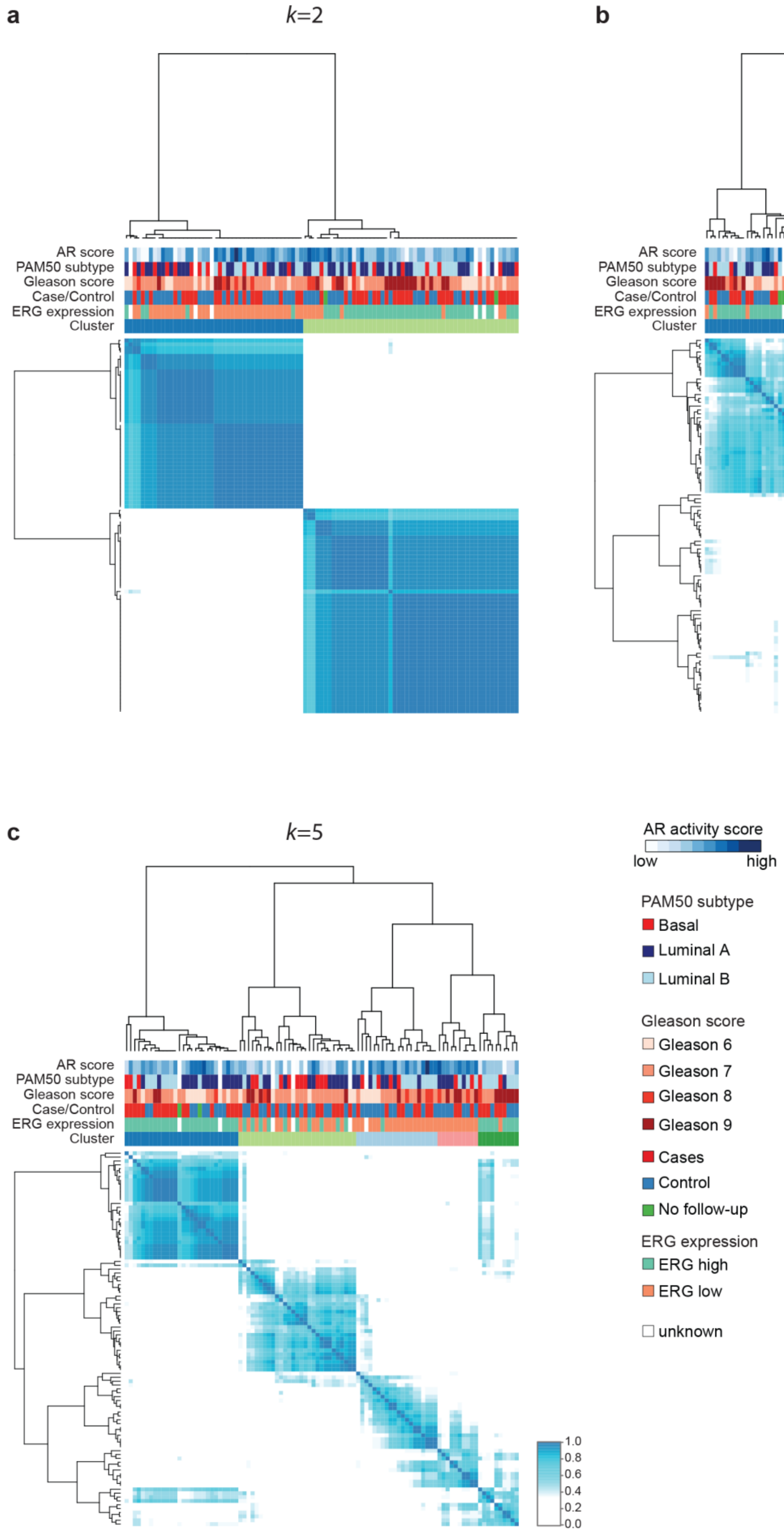
a
k=2
b
k=4
c
k=5
AR score
PAM50 subtype
Gleason score
Case/Control
ERG expression
Cluster
AR activity score
low
high
PAM50 subtype
Basal
Luminal A
Luminal B
Gleason score
Gleason 6
Gleason 7
Gleason 8
Gleason 9
Cases
Control
No follow-up
ERG expression
ERG high
ERG low
unknown
1.0
0.8
0.6
0.4
0.2
0.0

Supplementary Figure 12: Integrative clustering

Integrative cluster analysis using RNA-seq and ChIP-seq datasets for different cluster sizes of *k*=2 (**a**), *k*=4 (**b**) and *k*=5 (**c**). The darker the color blue, the higher the frequency of co-clustering of the samples. Each sample is annotated for AR activity score, PAM50 subtype, Gleason score, case/control status, ERG expression and cluster.

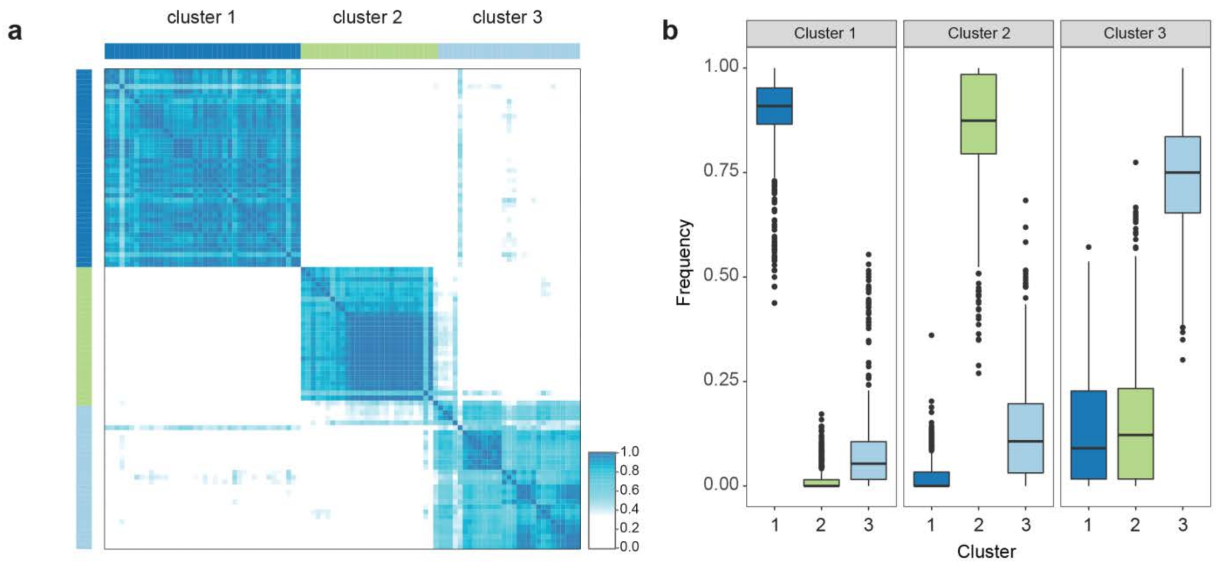

Supplementary Figure 13: Stability analysis of the three integrative clusters

- **a.** Heatmap shows frequency of sample pairs occurring in the same cluster in outcomes of 100 integrative clustering analysis with 80% subsampling. Rows and columns are samples, and the more frequently samples occur in the same cluster, the darker the color blue.
- **b.** Boxplots show stability of the three identified clusters. y axis is the frequency of classifying sample pairs in the same cluster and pairs are divided by final cluster assigned to the samples in pairs (x axis and three panels). Center line, median; box limits, upper (75) and lower (25) quartiles; whiskers, 1.5x interquartile range.

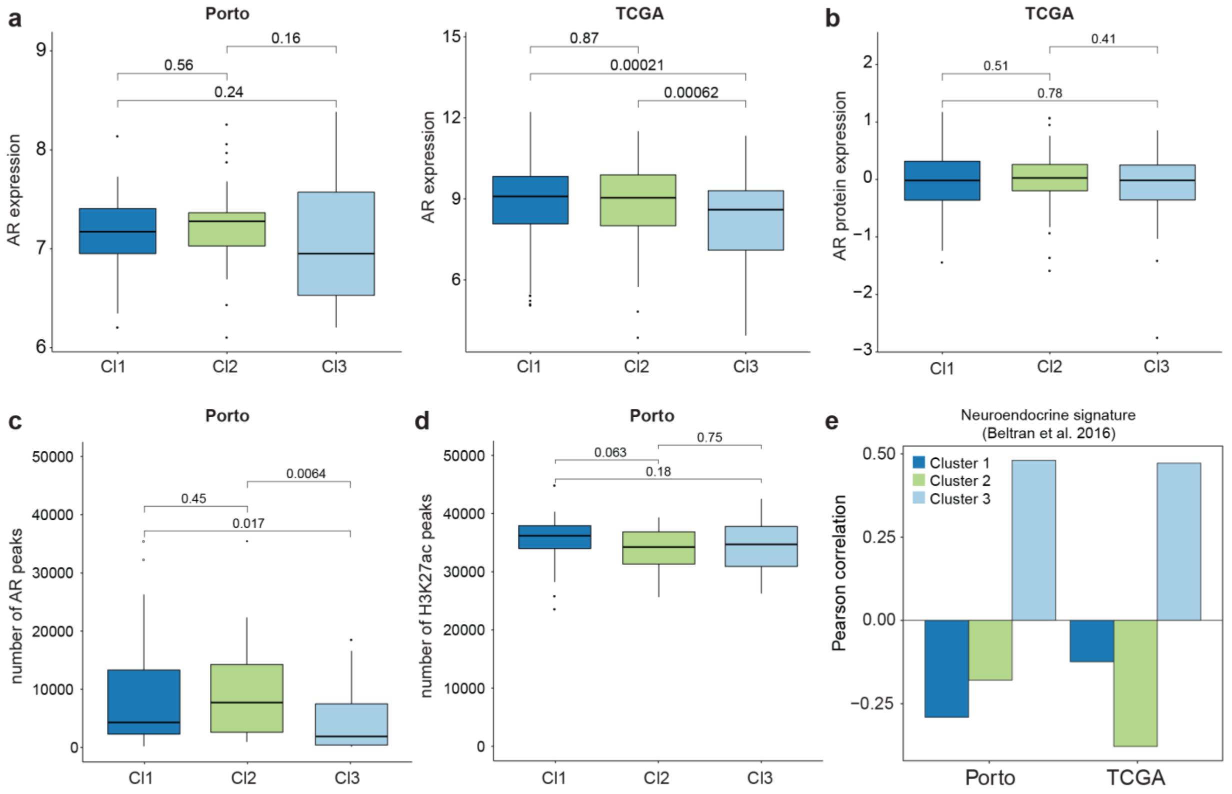

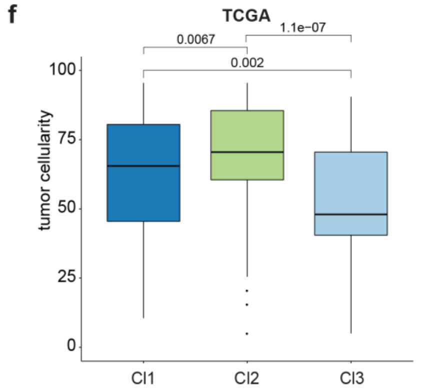

Supplementary Figure 14: Comparative analyses of the three prostate cancer subtypes

- **a.** Boxplot shows the log2 AR gene expression in the three clusters for the Porto (left) and TCGA (right) cohort. *p*-values were calculated using the Wilcoxon test. Center line, median; box limits, upper (75) and lower (25) quartiles; whiskers, 1.5x interquartile range.
- **b.** Boxplot shows the AR protein expression in TCGA (RPPA). *p*-values were calculated using the Wilcoxon test.
- **c.** Boxplot shows the number of AR peaks in the three clusters. *p*-values were calculated using the Wilcoxon test.
- **d.** Boxplot shows the number of H3K27ac peaks in the three clusters. *p*-values were calculated using the Wilcoxon test.
- **e.** Barplot shows the Pearson correlation of the neuroendocrine gene signature score in the three clusters for both the Porto and TCGA cohort.
- **f.** Boxplot shows the tumor percentage in the three clusters for the TCGA cohort. *p*-values were calculated using the Wilcoxon test.

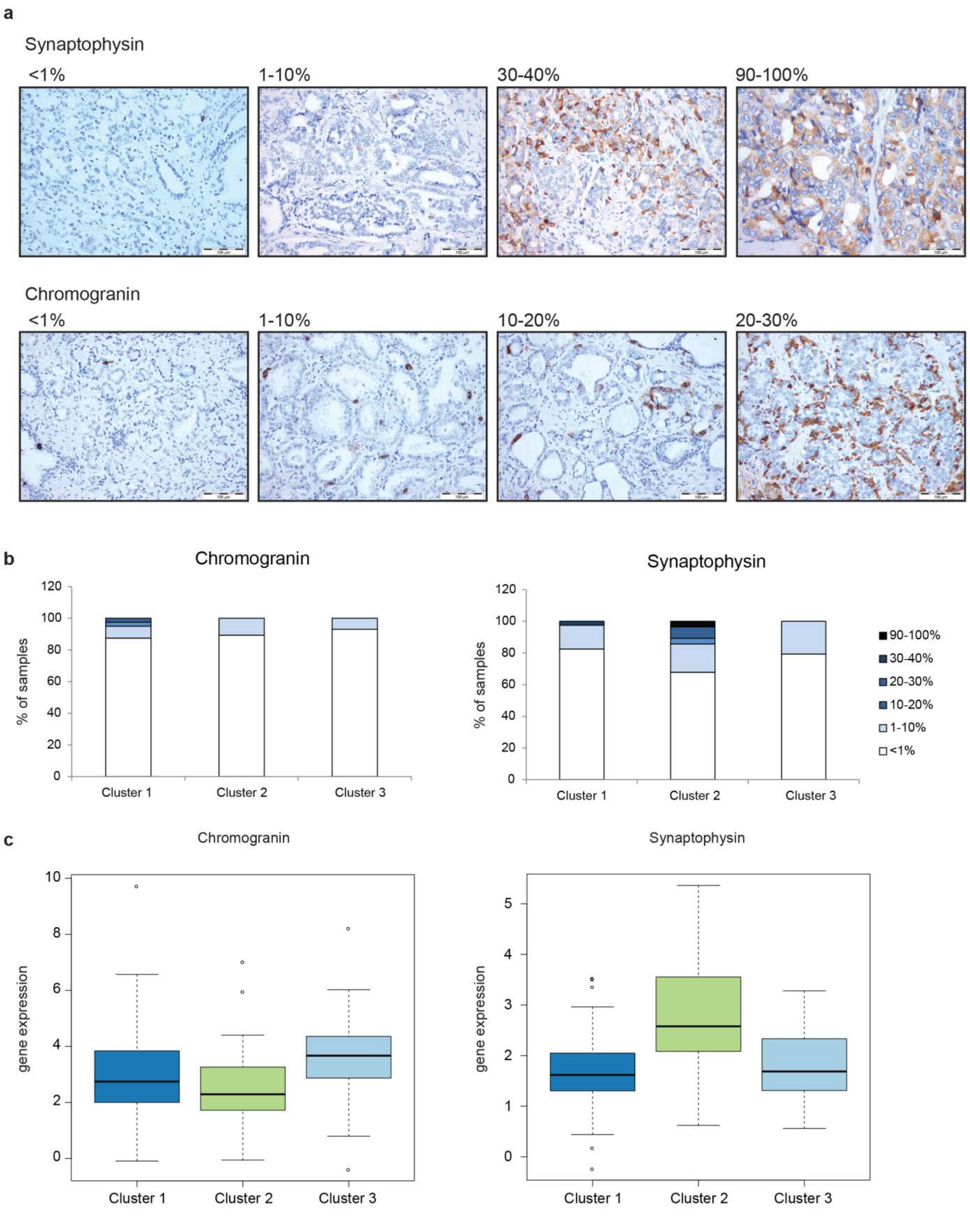

a
Synaptophysin
<1%
1-10%
30-40%
90-100%
Chromogranin
<1%
1-10%
10-20%
20-30%
b
Chromogranin
120
100
80
60
40
20
0
% of samples
Cluster 1
Cluster 2
Cluster 3
Synaptophysin
90-100%
30-40%
20-30%
10-20%
1-10%
<1%
c
Chromogranin
gene expression
10
8
6
4
2
0
Cluster 1
Cluster 2
Cluster 3
Synaptophysin
5
4
3
2
1
0

Supplementary Figure 15: Expression of neuroendocrine markers

a. Representative immunohistochemical staining for chromogranin and synaptophysin. Scale bars,100 µm.
b. Stacked bar charts comparing percentage of positive cells for chromogranin and synaptophysin expression across the three subtypes.
c. Boxplot shows the log2 chromogranin an synaptophysin gene expression in the three clusters.

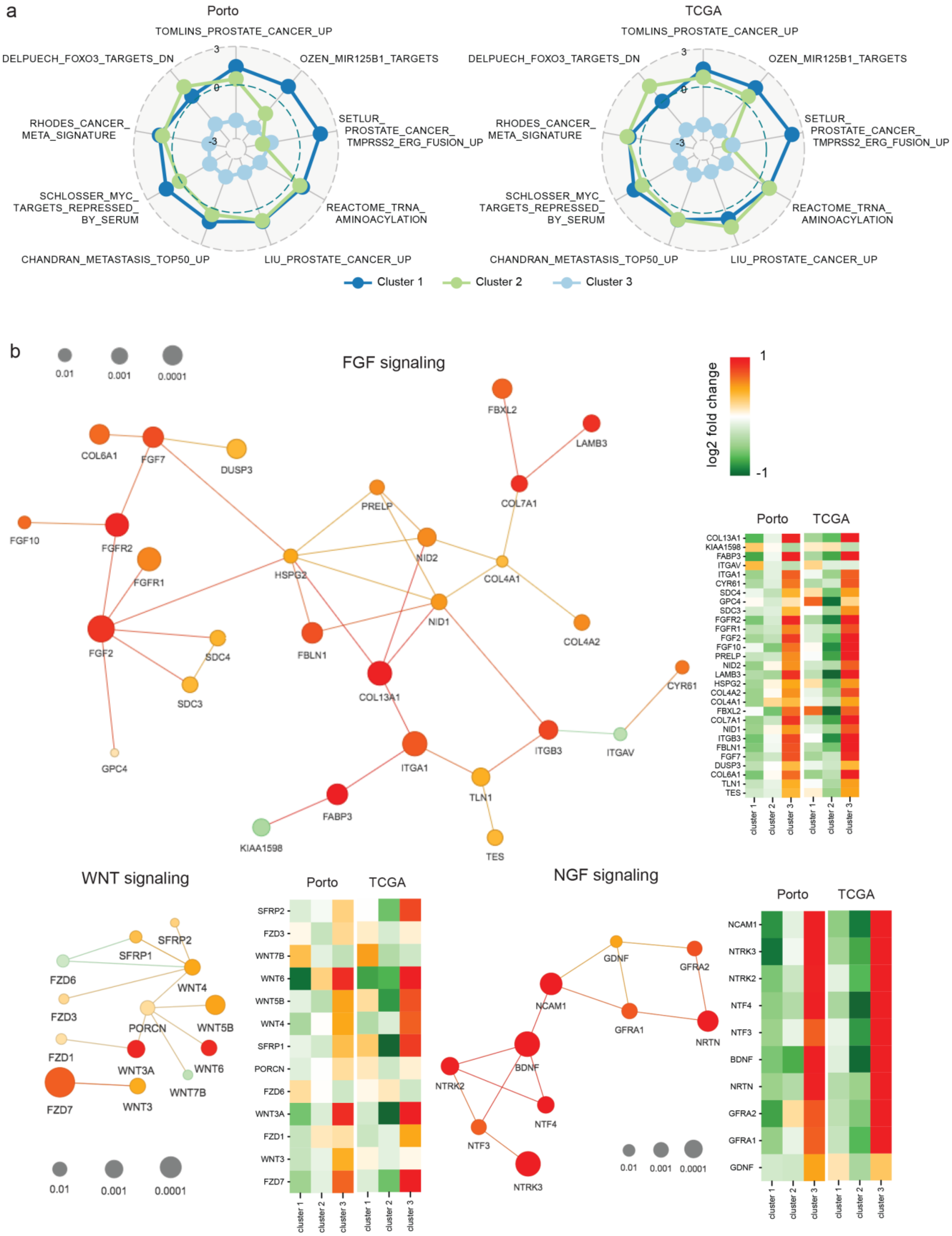

a
Porto
TCGA
TOMLINS_PROSTATE_CANCER_UP
OZEN_MIR125B1_TARGETS
SETLUR_PROSTATE_CANCER_TMPRSS2_ERG_FUSION_UP
REACTOME_TRNA_AMINOACYLATION
LIU_PROSTATE_CANCER_UP
CHANDRAN_METASTASIS_TOP50_UP
SCHLOSSER_MYC_TARGETS_REPRESSED_BY_SERUM
RHODES_CANCER_META_SIGNATURE
DELPUECH_FOXO3_TARGETS_DN
3
0
-3
Cluster 1
Cluster 2
Cluster 3
b
FGF signaling
0.01
0.001
0.0001
log2 fold change
1
-1
COL6A1
FGF7
DUSP3
FGF10
FGFR2
FGFR1
FGF2
SDC4
SDC3
GPC4
HSPG2
PRELP
NID2
NID1
COL4A1
COL4A2
FBXL2
LAMB3
COL7A1
FBLN1
COL13A1
ITGA1
ITGB3
ITGAV
CYR61
TLN1
TES
FABP3
KIAA1598
cluster 1
cluster 2
cluster 3
WNT signaling
SFRP2
SFRP1
FZD6
WNT4
FZD3
PORCN
WNT5B
FZD1
WNT3A
WNT6
WNT7B
FZD7
WNT3
WNT7B
WNT5B
NGF signaling
GDNF
GFRA2
NCAM1
GFRA1
NRTN
BDNF
NTRK2
NTF4
NTF3
NTRK3

Supplementary Figure 16: Gene expression-based characterization for the three prostate cancer subtypes

- **a.** Normalized enrichment scores (NES) for top gene sets enriched (FDR < 0.2) in any of the three clusters, represented in a radar plot for the Porto (left) and TCGA cohort (right). NES for each cluster is indicated with a line with the corresponding color.
- **b.** Differential regulatory networks identified by Hotnet2 for cluster 3. The size and color of nodes indicate FDR and log2 fold-change, respectively, obtained from differential gene expression analysis between cluster 3 relative to the other two. Log2 fold changes of the genes between the clusters obtained from both Porto and TCGA cohort are indicated in the heatmaps.
