# Supplementary material for: Integrative epigenetic taxonomy of primary prostate cancer
Source: Nat Commun. 2018 Nov 21;9:4900. doi: 10.1038/s41467-018-07270-2 (PMC6249266; doi:10.1038/s41467-018-07270-2)
Supplement: Supplementary file 3 — Description of Additional Supplementary Files [file 41467_2018_7270_MOESM3_ESM.pdf]

Description of Additional Supplementary Files

File Name: Supplementary Data 1
Description: ChIP-seq quality control metrics

File Name: Supplementary Data 2
Description: FRiP score and number of peaks from publicly available H3K27ac ChIP-seq samples

File Name: Supplementary Data 3
Description: List of 285 differentially expressed genes between cluster 1, 2 and 3

File Name: Supplementary Data 4
Description: List of significantly enriched gene sets

File Name: Supplementary Data 5.
Description: The eight largest networks enriched in cluster 3
