# Supplementary material for: Integrative epigenetic taxonomy of primary prostate cancer
Source: Nat Commun. 2018 Nov 21;9:4900. doi: 10.1038/s41467-018-07270-2 (PMC6249266; doi:10.1038/s41467-018-07270-2)
Supplement: Supplementary file 9 — Reporting Summary [file 41467_2018_7270_MOESM9_ESM.pdf]

nature research

Corresponding author(s): Wilbert Zwart
Andries M Bergman

# Reporting Summary

Nature Research wishes to improve the reproducibility of the work that we publish. This form provides structure for consistency and transparency in reporting. For further information on Nature Research policies, see Authors & Referees and the Editorial Policy Checklist.

## Statistical parameters

When statistical analyses are reported, confirm that the following items are present in the relevant location (e.g. figure legend, table legend, main text, or Methods section).

| n/a | Confirmed | |
|---|---|---|
| ☐ | ☒ | The exact sample size (*n*) for each experimental group/condition, given as a discrete number and unit of measurement |
| ☐ | ☒ | An indication of whether measurements were taken from distinct samples or whether the same sample was measured repeatedly |
| ☐ | ☒ | The statistical test(s) used AND whether they are one- or two-sided<br>*Only common tests should be described solely by name; describe more complex techniques in the Methods section.* |
| ☐ | ☒ | A description of all covariates tested |
| ☐ | ☒ | A description of any assumptions or corrections, such as tests of normality and adjustment for multiple comparisons |
| ☐ | ☒ | A full description of the statistics including central tendency (e.g. means) or other basic estimates (e.g. regression coefficient) AND variation (e.g. standard deviation) or associated estimates of uncertainty (e.g. confidence intervals) |
| ☐ | ☒ | For null hypothesis testing, the test statistic (e.g. *F*, *t*, *r*) with confidence intervals, effect sizes, degrees of freedom and *P* value noted<br>*Give P values as exact values whenever suitable.* |
| ☒ | ☐ | For Bayesian analysis, information on the choice of priors and Markov chain Monte Carlo settings |
| ☐ | ☒ | For hierarchical and complex designs, identification of the appropriate level for tests and full reporting of outcomes |
| ☐ | ☒ | Estimates of effect sizes (e.g. Cohen's *d*, Pearson's *r*), indicating how they were calculated |
| ☐ | ☒ | Clearly defined error bars<br>*State explicitly what error bars represent (e.g. SD, SE, CI)* |

*Our web collection on statistics for biologists may be useful.*

## Software and code

Policy information about availability of computer code

| | |
|---|---|
| Data collection | TCGA Biolinks was used to retrieve copy number data from the TCGA cohort. https://xenabrowser.net/ was used to retrieve gene expression, protein expression and mutation data from TCGA. NCBI GEO was used to obtain public AR ChIP -seq data and EGA to obtain public H3K27ac ChIP-seq data. All mentioned in the methods |
| Data analysis | All code is commercially available. Software versions are now updated in the manuscript: MACS2, MACS1.4, DFilter v1.5, ChromHMM (v1.12), phantompeaktools, BEDTools v2.25, BWA v0.5.10, TopHat, STAR fusion 0.5.4, MIV-NMF, EaSeq v1.03. The R packages DiffBind v2.4.6, ConsensusClusterPlus v1.40.01, CopywriteR v2.6.1, Circular Binary Segmentation (CBS), CGHcall, Limma v3.34, EdgeR v3.18.1 vand ggsea v1.0 were all used according to their manual. |

For manuscripts utilizing custom algorithms or software that are central to the research but not yet described in published literature, software must be made available to editors/reviewers upon request. We strongly encourage code deposition in a community repository (e.g. GitHub). See the Nature Research guidelines for submitting code & software for further information.

## Data

Policy information about availability of data

All manuscripts must include a data availability statement. This statement should provide the following information, where applicable:

- Accession codes, unique identifiers, or web links for publicly available datasets
- A list of figures that have associated raw data
- A description of any restrictions on data availability

In methods section, Data availability. ChIP-seq data generated in this study  to be filled in

# Field-specific reporting

Please select the best fit for your research. If you are not sure, read the appropriate sections before making your selection.

☒ Life sciences ☐ Behavioural & social sciences ☐ Ecological, evolutionary & environmental sciences

For a reference copy of the document with all sections, see nature.com/authors/policies/ReportingSummary-flat.pdf

# Life sciences study design

All studies must disclose on these points even when the disclosure is negative.

| | |
|---|---|
| Sample size | We attempted to obtain ChIP-seq data for 100 samples with known biochemical recurrence outcome. Our criteria were that the sample must have fresh frozen tissue available and high tumor cell percentage (see methods section, cohort). Data were successfully validated in the even larger cohort (TCGA). |
| Data exclusions | Data exclusion is reported in methods and visualized in supplementary figure 2 |
| Replication | To account for reproducibility we performed ChIP-seq in a large number of tissue samples (n=100).  In addition the gene expression profiles across the three subtypes were also found in the TCGA cohort (n=498). Reproducibility of the integrative clusters was succesfull as shown in supplementary figure 13 |
| Randomization | we allocated the samples into two groups based on the biochemical recurrence outcome of the patients. In the paper described as case and control groups |
| Blinding | For defining the case and control group, blinding was not possible, as we match the samples on clinical variables. The samples were blindly processed during sample processing. |

# Reporting for specific materials, systems and methods

## Materials & experimental systems

| n/a | Involved in the study | |
|---|---|---|
| ☒ | ☐ | Unique biological materials |
| ☐ | ☒ | Antibodies |
| ☒ | ☐ | Eukaryotic cell lines |
| ☒ | ☐ | Palaeontology |
| ☒ | ☐ | Animals and other organisms |
| ☒ | ☐ | Human research participants |

## Methods

| n/a | Involved in the study | |
|---|---|---|
| ☐ | ☒ | ChIP-seq |
| ☒ | ☐ | Flow cytometry |
| ☒ | ☐ | MRI-based neuroimaging |

## Antibodies

| | |
|---|---|
| Antibodies used | Antibodies used were AR (sc-816, Santa Cruz), H3K27ac (39133, Active Motif), H3K4me3 (Ab8580, Abcam) and H3K27me3 (39155, Active Motif). |
| Validation | The AR antibody is used in many ChIP-seq studies (e.g AR sc-816 in the study of Pomerantz et al 2015). The antibodies against the histone marks are used by ENCODE. |

# ChIP-seq

## Data deposition

☒ Confirm that both raw and final processed data have been deposited in a public database such as GEO.

☒ Confirm that you have deposited or provided access to graph files (e.g. BED files) for the called peaks.

| | |
|---|---|
| Data access links<br>*May remain private before publication.* | link to ChIP-seq:https://www.ncbi.nlm.nih.gov/geo/query/acc.cgi?acc=GSE120738<br>link to RNA-seq:https://www.ncbi.nlm.nih.gov/geo/query/acc.cgi?acc=GSE120741 |
| Files in database submission | *Provide a list of all files available in the database submission.* |
| Genome browser session<br>(e.g. UCSC) | *Provide a link to an anonymized genome browser session for "Initial submission" and "Revised version" documents only, to enable peer review. Write "no longer applicable" for "Final submission" documents.* |

## Methodology

| | |
|---|---|
| Replicates | 100 prostate cancer samples for ChIP-seq and RNA-seq |
| Sequencing depth | Single end read sequencing (65bp). We aimed at sequencing 20 million reads per sample for both RNA and ChIP-seq. The total number of reads, number of aligned reads and other sequencing parameters are listed in table S1 and visualized in supplementary figure 3 |
| Antibodies | Antibodies used were AR (sc-816, Santa Cruz), H3K27ac (39133, Active Motif), H3K4me3 (Ab8580, Abcam) and H3K27me3 (39155, Active Motif). |
| Peak calling parameters | Peak calling over input control (mixed inputs) was performed using DFilter and MACS for AR and H3K27ac ChIP-seq samples. MACS 1.4 was run with p-value cutoff of 10e-7 and DFilter with bs=50, ks=30, refine, nonzero. For H3K4me3, MACS2 and DFilter were used with broad-peak settings: 1) –broad and –broad-cutoff=0.2 for MACS2 and 2) bs=100 and ks=60 for DFilter. The peaks called by both peak callers were used for analysis. H3K27me3 ChIP-seq peaks were called by genome segmentation using ChromHMM chosing the state with high H3K27me3 signal. |
| Data quality | The number of peaks and quality measures are reported in Table S1. |
| Software | Genome browser snapshots were generated using Easeq, motif analysis was performed using the Galaxy Cistrome SeqPos motif tool with default settings and genomic region enrichment analysis was performed with CEAS. Consensus peaklists were generated with the DiffBind R package. BEDTools was used to calculated read counts in peaks. The raw counts were normalized for library size followed by TMM normalization using EdgeR. |
